# 4-[(5-Bromo-2-hy­droxy­benzyl­idene)amino]-3-propyl-1*H*-1,2,4-triazole-5(4*H*)-thione

**DOI:** 10.1107/S1600536812019745

**Published:** 2012-05-12

**Authors:** Xin Wu, Cai-Xia Yuan, Ling Ma, Kai-Lu Zhai, Miao-Li Zhu

**Affiliations:** a College of Arts and Sciences, Shanxi Agricultural University, Taigu, Shanxi 030801, People’s Republic of China; b Institute of Molecular Science, Key Laboratory of Chemical Biology and Molecular Engineering of the Education Ministry, Shanxi University, Taiyuan, Shanxi 030006, People’s Republic of China; c Department of Biochemistry and Molecular Biology, Shanxi Medical University, Taiyuan, Shanxi 030001, People’s Republic of China; d Jincheng Tap Water Company, Jincheng, Shanxi 048000, People’s Republic of China

## Abstract

The asymmetric unit of the title compound, C_12_H_13_BrN_4_OS, contains two independent mol­ecules in which the dihedral angles between the triazole and benzene rings are 2.9 (3) and 7.5 (3)°. The thione group is of the form *R*_2_C=S. An intra­molecular O—H⋯N hydrogen bond occurs in each mol­ecule. The crystal structure features weak N—H⋯S inter­actions and π–π stacking of the benzene rings [centroid–centroid distance = 3.667 (3) Å].

## Related literature

For the pharmacological activity of 1,2,4-triazole-substituted and Schiff base compounds, see: Isloor *et al.* (2009 ▶); Ma *et al.* (2011 ▶). For copper complexes containing 1,2,4-triazole Schiff base ligands, see: Wen *et al.* (2004 ▶).
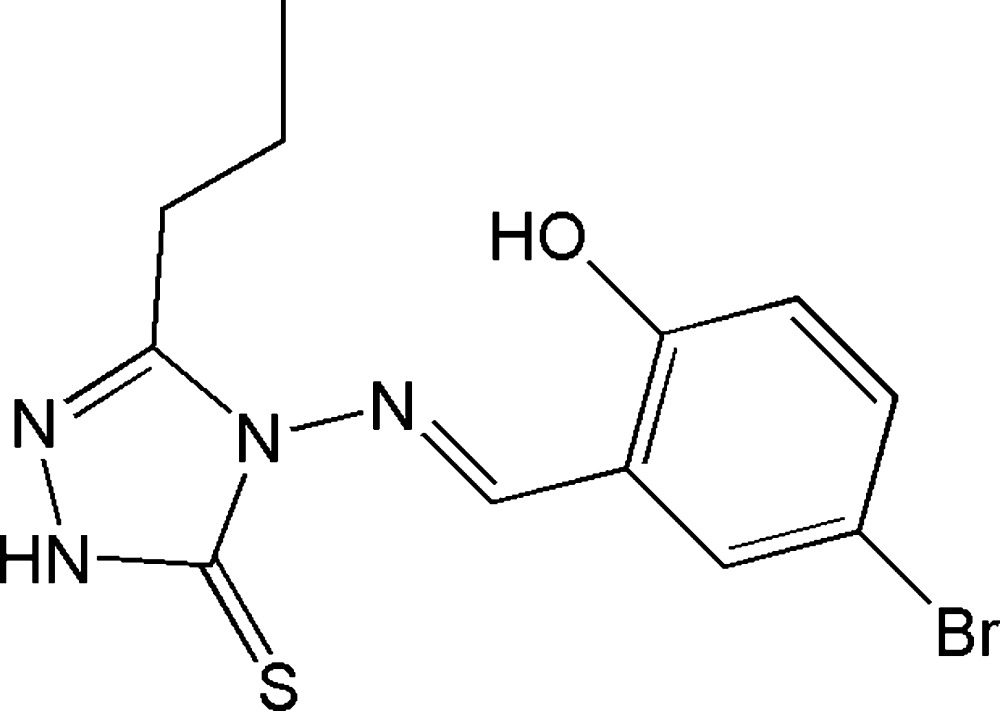


## Experimental

### 

#### Crystal data

C_12_H_13_BrN_4_OS
*M* *_r_* = 341.23
Triclinic, 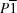

*a* = 8.042 (5) Å
*b* = 13.187 (8) Å
*c* = 13.408 (8) Å
α = 97.406 (9)°
β = 92.956 (10)°
γ = 95.916 (9)°
*V* = 1399.5 (15) Å^3^
*Z* = 4
Mo *K*α radiation
μ = 3.08 mm^−1^
*T* = 298 K
0.30 × 0.25 × 0.20 mm

#### Data collection

Bruker SMART 1K CCD diffractometer
Absorption correction: multi-scan (*SADABS*; Sheldrick, 2000 ▶) *T*_min_ = 0.458, *T*_max_ = 0.578
14278 measured reflections
4939 independent reflections
3061 reflections with *I* > 2σ(*I*)
*R*_int_ = 0.055

#### Refinement

*R*[*F* ^2^ > 2σ(*F* ^2^)] = 0.043
*wR*(*F* ^2^) = 0.102
*S* = 0.93
4939 reflections
347 parameters
H-atom parameters constrained
Δρ_max_ = 0.49 e Å^−3^
Δρ_min_ = −0.55 e Å^−3^

### 

Data collection: *SMART* (Bruker, 2000 ▶); cell refinement: *SAINT* (Bruker, 2000 ▶); data reduction: *SAINT*; program(s) used to solve structure: *SHELXS97* (Sheldrick, 2008 ▶); program(s) used to refine structure: *SHELXL97* (Sheldrick, 2008 ▶); molecular graphics: *ORTEP-3* (Farrugia, 1997 ▶); software used to prepare material for publication: *publCIF* (Westrip, 2010 ▶).

## Supplementary Material

Crystal structure: contains datablock(s) I, global. DOI: 10.1107/S1600536812019745/jj2128sup1.cif

Structure factors: contains datablock(s) I. DOI: 10.1107/S1600536812019745/jj2128Isup2.hkl

Supplementary material file. DOI: 10.1107/S1600536812019745/jj2128Isup3.cml

Additional supplementary materials:  crystallographic information; 3D view; checkCIF report

## Figures and Tables

**Table 1:** Hydrogen-bond geometry (Å, °)

*D*—H⋯*A*	*D*—H	H⋯*A*	*D*⋯*A*	*D*—H⋯*A*
O1—H1⋯N4	0.82	1.93	2.644 (4)	145
O2—H2⋯N8	0.82	1.90	2.612 (4)	144
N1—H1*A*⋯S1^i^	0.86	2.46	3.300 (4)	164
N5—H5⋯S2^ii^	0.86	2.41	3.252 (4)	165

Symmetry codes: (i) 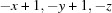; (ii) 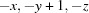.

**Fig. 1.**
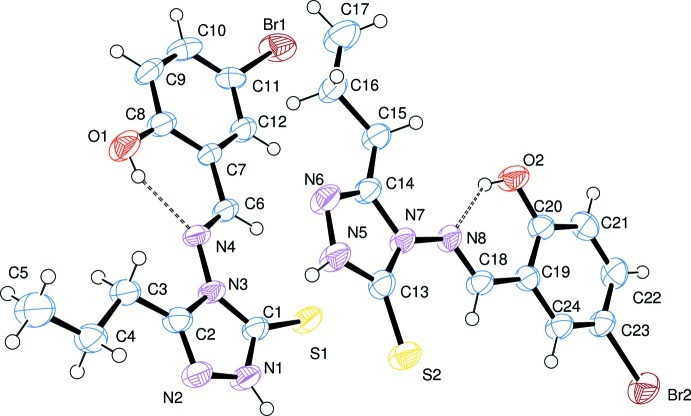
Molecular structure of the title compound, (I), with displacement ellipsoids drawn at the 50% probability level. Dashed lines indicate O—H···N intramolecular hydrogen bonding.

**Fig. 2.**
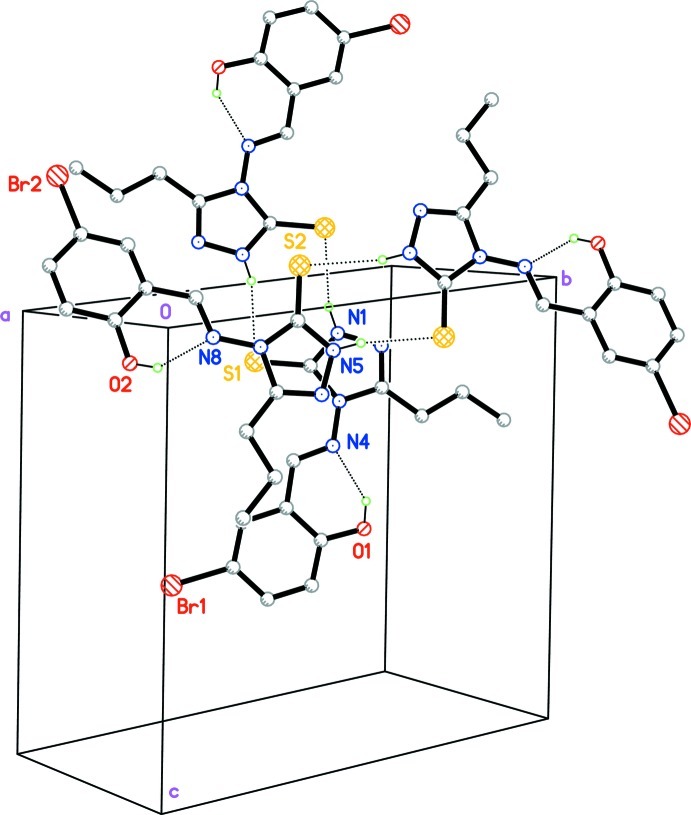
Packing of the title compound, (I),viewed along the aaxis. Dashed lines indicate O—H···N intramolecular hydrogen bonds and weak N—H···S intermolecular interactions. Remaining hydrogen atoms have been removed for clarity.

## supplementary crystallographic information

### Comment

1,2,4-triazole and its derivatives possess a variety of pharmacological properties (Isloor *et al.*, 2009). Schiff bases derived from substituted salicylaldehydes form numerous metal complexes (Wen *et al.*, 2004). Also, copper complexes containing 1,2,4-triazole Schiff base ligands are potential inhibitors of PTP1B (protein tyrosine phosphatase 1B), TCPTP (T-cell protein tyrosine phosphatase), PTP-MEG2 (megakaryocyte protein tyrosine phosphatase) and SHP-1 (Src homology phosphatase 1) (Ma *et al.*, 2011). In continuation of our work in this area, we report here the synthesis and crystal structure of the title compound, C_12_H_13_BrN_4_OS, (I).

The title compound, (I), crystallizes with two independent molecules in the asymmetric unit (Fig. 1). The dihedral angles between the triazole and phenyl rings are 2.9 (3)° and 7.5 (3)°, respectively. The thione group is of the form *R*_2_C=S with a Cdb\S distance of 1.673 (4)Å and 1.675 (4)Å indicating significant double bond character. Intramolecular O—H···N hydrogen bonds, weak N—H···S intermolecular interactions (Table 1) and π–π stacking of the benzene rings [centroid–centroid distance = 3.667 (3) Å] are observed that may influence crystal packing.

### Experimental

0.5 mmol 5-Bromosalicylaldehyde in 10 ml of ethanol was added to a solution of 4-amino-5-propyl-1,2,4-triazole-3-thione (0.5 mmol) in 20 ml of ethanol, and then refluxed for 2 h. The resulting solution was filterd and recrystallized from ethanol. X-ray quality Yellow crystals of the title compound were formed upon slow evaporation of the resulting soluton.

### Refinement

All of the H atoms were placed in their calculated positions and the refined using the riding model with C—H lengths of 0.93Å (CH), 0.97Å (CH_2_), 0.97 Å (CH_3_), 0.86 (NH) or 0.82Å. The isotropoc displacement parameters for these atoms were set to 1.2 (CH, CH_2_, O, N) or 1.5 (CH_3_) times *U*_eq_ of the parent atom.

### Crystal data

**Table:**

C_12_H_13_BrN_4_OS	*Z* = 4
*M**_r_* = 341.23	*F*(000) = 688
Triclinic, *P*1	*D*_x_ = 1.620 Mg m^−^^3^
Hall symbol: -P 1	Mo *K*α radiation, λ = 0.71073 Å
*a* = 8.042 (5) Å	Cell parameters from 2374 reflections
*b* = 13.187 (8) Å	θ = 2.5–23.6°
*c* = 13.408 (8) Å	µ = 3.08 mm^−^^1^
α = 97.406 (9)°	*T* = 298 K
β = 92.956 (10)°	Block, yellow
γ = 95.916 (9)°	0.30 × 0.25 × 0.20 mm
*V* = 1399.5 (15) Å^3^	

### Data collection

**Table:**

Bruker SMART 1K CCD diffractometer	4939 independent reflections
Radiation source: fine-focus sealed tube	3061 reflections with *I* > 2σ(*I*)
Graphite monochromator	*R*_int_ = 0.055
Detector resolution: 11.72 pixels mm^-1^	θ_max_ = 25.0°, θ_min_ = 1.5°
ω scans	*h* = −9→9
Absorption correction: multi-scan (*SADABS*; Sheldrick, 2000)	*k* = −15→15
*T*_min_ = 0.458, *T*_max_ = 0.578	*l* = −15→15
14278 measured reflections	

### Refinement

**Table:**

Refinement on *F*^2^	Primary atom site location: structure-invariant direct methods
Least-squares matrix: full	Secondary atom site location: difference Fourier map
*R*[*F*^2^ > 2σ(*F*^2^)] = 0.043	Hydrogen site location: inferred from neighbouring sites
*wR*(*F*^2^) = 0.102	H-atom parameters constrained
*S* = 0.93	*w* = 1/[σ^2^(*F*_o_^2^) + (0.0516*P*)^2^] where *P* = (*F*_o_^2^ + 2*F*_c_^2^)/3
4939 reflections	(Δ/σ)_max_ < 0.001
347 parameters	Δρ_max_ = 0.49 e Å^−^^3^
0 restraints	Δρ_min_ = −0.55 e Å^−^^3^

### Special details

**Table:**

Geometry. All e.s.d.'s (except the e.s.d. in the dihedral angle between two l.s. planes) are estimated using the full covariance matrix. The cell e.s.d.'s are taken into account individually in the estimation of e.s.d.'s in distances, angles and torsion angles; correlations between e.s.d.'s in cell parameters are only used when they are defined by crystal symmetry. An approximate (isotropic) treatment of cell e.s.d.'s is used for estimating e.s.d.'s involving l.s. planes.
Refinement. Refinement of *F*^2^ against ALL reflections. The weighted *R*-factor *wR* and goodness of fit *S* are based on *F*^2^, conventional *R*-factors *R* are based on *F*, with *F* set to zero for negative *F*^2^. The threshold expression of *F*^2^ > σ(*F*^2^) is used only for calculating *R*-factors(gt) *etc*. and is not relevant to the choice of reflections for refinement. *R*-factors based on *F*^2^ are statistically about twice as large as those based on *F*, and *R*- factors based on ALL data will be even larger.

### Fractional atomic coordinates and isotropic or equivalent isotropic displacement parameters (Å^2^)

**Table:**

	*x*	*y*	*z*	*U*_iso_*/*U*_eq_	
Br1	0.38916 (7)	0.16296 (4)	0.59088 (4)	0.06736 (19)	
S1	0.49656 (16)	0.40893 (8)	0.14062 (8)	0.0530 (3)	
O1	0.1987 (5)	0.5771 (2)	0.5179 (2)	0.0666 (10)	
H1	0.2089	0.5860	0.4590	0.100*	
N1	0.4061 (5)	0.5891 (3)	0.0953 (2)	0.0521 (10)	
H1A	0.4451	0.5805	0.0366	0.062*	
N2	0.3314 (5)	0.6750 (3)	0.1302 (3)	0.0512 (10)	
N3	0.3366 (4)	0.5663 (2)	0.2414 (2)	0.0378 (8)	
N4	0.3044 (4)	0.5369 (2)	0.3350 (2)	0.0400 (8)	
C1	0.4132 (5)	0.5203 (3)	0.1597 (3)	0.0389 (10)	
C2	0.2882 (5)	0.6584 (3)	0.2197 (3)	0.0424 (10)	
C3	0.1971 (6)	0.7272 (3)	0.2879 (3)	0.0462 (11)	
H3A	0.2588	0.7428	0.3531	0.055*	
H3B	0.0881	0.6922	0.2975	0.055*	
C4	0.1740 (6)	0.8271 (3)	0.2457 (3)	0.0540 (12)	
H4A	0.2832	0.8624	0.2370	0.065*	
H4B	0.1143	0.8111	0.1799	0.065*	
C5	0.0785 (7)	0.8980 (4)	0.3133 (4)	0.0833 (18)	
H5A	−0.0316	0.8647	0.3197	0.125*	
H5B	0.0695	0.9604	0.2844	0.125*	
H5C	0.1368	0.9140	0.3786	0.125*	
C6	0.3493 (5)	0.4529 (3)	0.3590 (3)	0.0420 (11)	
H6	0.4027	0.4104	0.3131	0.050*	
C7	0.3181 (5)	0.4230 (3)	0.4566 (3)	0.0368 (10)	
C8	0.2452 (5)	0.4844 (3)	0.5321 (3)	0.0466 (11)	
C9	0.2203 (6)	0.4511 (4)	0.6243 (3)	0.0554 (13)	
H9	0.1734	0.4926	0.6743	0.066*	
C10	0.2647 (6)	0.3566 (4)	0.6423 (3)	0.0562 (13)	
H10	0.2475	0.3341	0.7043	0.067*	
C11	0.3349 (5)	0.2955 (3)	0.5681 (3)	0.0458 (11)	
C12	0.3633 (5)	0.3282 (3)	0.4773 (3)	0.0442 (11)	
H12	0.4133	0.2868	0.4287	0.053*	
Br2	0.51614 (6)	−0.08187 (4)	−0.31301 (4)	0.06348 (19)	
S2	0.14032 (16)	0.37417 (8)	−0.08568 (8)	0.0509 (3)	
O2	0.1831 (4)	−0.0214 (2)	0.0781 (2)	0.0544 (8)	
H2	0.1680	0.0389	0.0943	0.082*	
N6	−0.0208 (5)	0.3668 (3)	0.1823 (2)	0.0499 (9)	
N5	0.0102 (5)	0.4074 (2)	0.0951 (2)	0.0516 (10)	
H5	−0.0145	0.4674	0.0853	0.062*	
N7	0.0963 (4)	0.2607 (2)	0.0741 (2)	0.0374 (8)	
N8	0.1549 (4)	0.1671 (2)	0.0448 (2)	0.0385 (8)	
C13	0.0814 (5)	0.3467 (3)	0.0267 (3)	0.0418 (10)	
C14	0.0324 (5)	0.2768 (3)	0.1686 (3)	0.0413 (10)	
C15	0.0280 (5)	0.2020 (3)	0.2411 (3)	0.0441 (11)	
H15A	0.1416	0.1892	0.2590	0.053*	
H15B	−0.0325	0.1375	0.2094	0.053*	
C16	−0.0556 (6)	0.2392 (3)	0.3367 (3)	0.0495 (12)	
H16A	0.0035	0.3043	0.3679	0.059*	
H16B	−0.1700	0.2505	0.3191	0.059*	
C17	−0.0559 (7)	0.1630 (4)	0.4107 (3)	0.0694 (15)	
H17A	−0.1174	0.0991	0.3808	0.104*	
H17B	−0.1078	0.1893	0.4701	0.104*	
H17C	0.0573	0.1519	0.4285	0.104*	
C18	0.2235 (5)	0.1495 (3)	−0.0384 (3)	0.0414 (10)	
H18	0.2378	0.2006	−0.0799	0.050*	
C19	0.2791 (5)	0.0495 (3)	−0.0681 (3)	0.0368 (10)	
C20	0.2579 (5)	−0.0309 (3)	−0.0101 (3)	0.0395 (10)	
C21	0.3146 (6)	−0.1252 (3)	−0.0421 (3)	0.0497 (12)	
H21	0.3009	−0.1784	−0.0030	0.060*	
C22	0.3909 (5)	−0.1398 (3)	−0.1314 (3)	0.0490 (11)	
H22	0.4284	−0.2029	−0.1529	0.059*	
C23	0.4117 (5)	−0.0604 (3)	−0.1893 (3)	0.0428 (10)	
C24	0.3565 (5)	0.0322 (3)	−0.1588 (3)	0.0427 (11)	
H24	0.3704	0.0846	−0.1989	0.051*	

### Atomic displacement parameters (Å^2^)

**Table:**

	*U*^11^	*U*^22^	*U*^33^	*U*^12^	*U*^13^	*U*^23^
Br1	0.0834 (4)	0.0674 (3)	0.0629 (3)	0.0222 (3)	0.0151 (3)	0.0374 (3)
S1	0.0863 (9)	0.0448 (7)	0.0363 (6)	0.0296 (6)	0.0219 (6)	0.0124 (5)
O1	0.104 (3)	0.053 (2)	0.053 (2)	0.0347 (19)	0.038 (2)	0.0096 (16)
N1	0.081 (3)	0.045 (2)	0.039 (2)	0.027 (2)	0.028 (2)	0.0140 (17)
N2	0.076 (3)	0.042 (2)	0.043 (2)	0.0232 (19)	0.025 (2)	0.0148 (17)
N3	0.054 (2)	0.0365 (19)	0.0274 (18)	0.0125 (16)	0.0092 (16)	0.0128 (14)
N4	0.052 (2)	0.040 (2)	0.0313 (19)	0.0078 (17)	0.0147 (17)	0.0110 (15)
C1	0.052 (3)	0.039 (2)	0.030 (2)	0.011 (2)	0.008 (2)	0.0099 (18)
C2	0.048 (3)	0.043 (2)	0.040 (2)	0.011 (2)	0.010 (2)	0.0129 (19)
C3	0.057 (3)	0.039 (2)	0.049 (3)	0.018 (2)	0.021 (2)	0.015 (2)
C4	0.072 (3)	0.045 (3)	0.051 (3)	0.019 (2)	0.018 (3)	0.017 (2)
C5	0.115 (5)	0.056 (3)	0.097 (4)	0.045 (3)	0.057 (4)	0.028 (3)
C6	0.061 (3)	0.037 (2)	0.031 (2)	0.010 (2)	0.016 (2)	0.0079 (18)
C7	0.047 (3)	0.038 (2)	0.025 (2)	0.0033 (19)	0.0080 (19)	0.0050 (17)
C8	0.052 (3)	0.051 (3)	0.039 (3)	0.009 (2)	0.015 (2)	0.009 (2)
C9	0.074 (4)	0.061 (3)	0.034 (3)	0.013 (3)	0.023 (2)	0.003 (2)
C10	0.062 (3)	0.078 (4)	0.034 (3)	0.008 (3)	0.014 (2)	0.022 (2)
C11	0.052 (3)	0.049 (3)	0.040 (2)	0.004 (2)	0.009 (2)	0.020 (2)
C12	0.060 (3)	0.043 (2)	0.032 (2)	0.009 (2)	0.009 (2)	0.0082 (19)
Br2	0.0799 (4)	0.0558 (3)	0.0581 (3)	0.0235 (3)	0.0309 (3)	−0.0026 (2)
S2	0.0755 (9)	0.0429 (6)	0.0419 (6)	0.0249 (6)	0.0211 (6)	0.0133 (5)
O2	0.085 (2)	0.0426 (17)	0.0434 (18)	0.0200 (17)	0.0240 (17)	0.0158 (14)
N6	0.068 (3)	0.049 (2)	0.037 (2)	0.0223 (19)	0.0166 (19)	0.0066 (17)
N5	0.083 (3)	0.039 (2)	0.041 (2)	0.0294 (19)	0.023 (2)	0.0109 (16)
N7	0.051 (2)	0.0321 (18)	0.0332 (18)	0.0170 (16)	0.0127 (16)	0.0046 (14)
N8	0.051 (2)	0.0298 (18)	0.0369 (19)	0.0156 (16)	0.0104 (17)	0.0020 (14)
C13	0.055 (3)	0.034 (2)	0.038 (2)	0.015 (2)	0.007 (2)	0.0039 (18)
C14	0.049 (3)	0.041 (3)	0.036 (2)	0.013 (2)	0.013 (2)	0.0047 (19)
C15	0.055 (3)	0.044 (2)	0.037 (2)	0.015 (2)	0.010 (2)	0.0093 (19)
C16	0.063 (3)	0.053 (3)	0.034 (2)	0.015 (2)	0.014 (2)	0.002 (2)
C17	0.097 (4)	0.076 (4)	0.042 (3)	0.023 (3)	0.015 (3)	0.019 (3)
C18	0.055 (3)	0.036 (2)	0.037 (2)	0.014 (2)	0.014 (2)	0.0087 (18)
C19	0.044 (3)	0.031 (2)	0.037 (2)	0.0097 (19)	0.005 (2)	0.0057 (18)
C20	0.046 (3)	0.036 (2)	0.039 (2)	0.010 (2)	0.006 (2)	0.0096 (19)
C21	0.067 (3)	0.030 (2)	0.055 (3)	0.010 (2)	0.004 (3)	0.013 (2)
C22	0.060 (3)	0.037 (2)	0.053 (3)	0.021 (2)	0.007 (2)	0.001 (2)
C23	0.047 (3)	0.042 (2)	0.041 (2)	0.013 (2)	0.013 (2)	0.0011 (19)
C24	0.055 (3)	0.039 (2)	0.039 (2)	0.017 (2)	0.014 (2)	0.0085 (19)

### Geometric parameters (Å, º)

**Table:**

Br1—C11	1.902 (4)	Br2—C23	1.900 (4)
S1—C1	1.673 (4)	S2—C13	1.675 (4)
O1—C8	1.348 (5)	O2—C20	1.351 (4)
O1—H1	0.8200	O2—H2	0.8200
N1—C1	1.334 (5)	N6—C14	1.297 (5)
N1—N2	1.377 (4)	N6—N5	1.371 (4)
N1—H1A	0.8600	N5—C13	1.330 (5)
N2—C2	1.304 (5)	N5—H5	0.8600
N3—C2	1.375 (5)	N7—C13	1.381 (5)
N3—C1	1.385 (5)	N7—N8	1.384 (4)
N3—N4	1.390 (4)	N7—C14	1.391 (5)
N4—C6	1.274 (5)	N8—C18	1.274 (4)
C2—C3	1.479 (5)	C14—C15	1.470 (5)
C3—C4	1.524 (5)	C15—C16	1.527 (5)
C3—H3A	0.9700	C15—H15A	0.9700
C3—H3B	0.9700	C15—H15B	0.9700
C4—C5	1.512 (6)	C16—C17	1.500 (6)
C4—H4A	0.9700	C16—H16A	0.9700
C4—H4B	0.9700	C16—H16B	0.9700
C5—H5A	0.9600	C17—H17A	0.9600
C5—H5B	0.9600	C17—H17B	0.9600
C5—H5C	0.9600	C17—H17C	0.9600
C6—C7	1.442 (5)	C18—C19	1.450 (5)
C6—H6	0.9300	C18—H18	0.9300
C7—C12	1.394 (5)	C19—C20	1.395 (5)
C7—C8	1.404 (5)	C19—C24	1.398 (5)
C8—C9	1.381 (5)	C20—C21	1.392 (5)
C9—C10	1.378 (6)	C21—C22	1.374 (6)
C9—H9	0.9300	C21—H21	0.9300
C10—C11	1.380 (6)	C22—C23	1.383 (6)
C10—H10	0.9300	C22—H22	0.9300
C11—C12	1.366 (5)	C23—C24	1.363 (5)
C12—H12	0.9300	C24—H24	0.9300
			
C8—O1—H1	109.5	C20—O2—H2	109.5
C1—N1—N2	114.6 (3)	C14—N6—N5	104.5 (3)
C1—N1—H1A	122.7	C13—N5—N6	114.8 (3)
N2—N1—H1A	122.7	C13—N5—H5	122.6
C2—N2—N1	104.0 (3)	N6—N5—H5	122.6
C2—N3—C1	109.1 (3)	C13—N7—N8	133.1 (3)
C2—N3—N4	118.4 (3)	C13—N7—C14	108.8 (3)
C1—N3—N4	132.6 (3)	N8—N7—C14	118.0 (3)
C6—N4—N3	120.6 (3)	C18—N8—N7	121.2 (3)
N1—C1—N3	102.0 (3)	N5—C13—N7	102.2 (3)
N1—C1—S1	126.4 (3)	N5—C13—S2	126.8 (3)
N3—C1—S1	131.5 (3)	N7—C13—S2	131.0 (3)
N2—C2—N3	110.3 (3)	N6—C14—N7	109.7 (3)
N2—C2—C3	125.3 (4)	N6—C14—C15	125.9 (3)
N3—C2—C3	124.4 (3)	N7—C14—C15	124.3 (3)
C2—C3—C4	111.6 (3)	C14—C15—C16	112.5 (3)
C2—C3—H3A	109.3	C14—C15—H15A	109.1
C4—C3—H3A	109.3	C16—C15—H15A	109.1
C2—C3—H3B	109.3	C14—C15—H15B	109.1
C4—C3—H3B	109.3	C16—C15—H15B	109.1
H3A—C3—H3B	108.0	H15A—C15—H15B	107.8
C5—C4—C3	112.6 (3)	C17—C16—C15	111.8 (3)
C5—C4—H4A	109.1	C17—C16—H16A	109.3
C3—C4—H4A	109.1	C15—C16—H16A	109.3
C5—C4—H4B	109.1	C17—C16—H16B	109.3
C3—C4—H4B	109.1	C15—C16—H16B	109.3
H4A—C4—H4B	107.8	H16A—C16—H16B	107.9
C4—C5—H5A	109.5	C16—C17—H17A	109.5
C4—C5—H5B	109.5	C16—C17—H17B	109.5
H5A—C5—H5B	109.5	H17A—C17—H17B	109.5
C4—C5—H5C	109.5	C16—C17—H17C	109.5
H5A—C5—H5C	109.5	H17A—C17—H17C	109.5
H5B—C5—H5C	109.5	H17B—C17—H17C	109.5
N4—C6—C7	120.7 (4)	N8—C18—C19	119.7 (3)
N4—C6—H6	119.7	N8—C18—H18	120.1
C7—C6—H6	119.7	C19—C18—H18	120.1
C12—C7—C8	118.3 (4)	C20—C19—C24	118.3 (3)
C12—C7—C6	118.2 (3)	C20—C19—C18	122.8 (3)
C8—C7—C6	123.5 (4)	C24—C19—C18	118.9 (3)
O1—C8—C9	118.0 (4)	O2—C20—C21	117.4 (3)
O1—C8—C7	121.8 (4)	O2—C20—C19	122.4 (3)
C9—C8—C7	120.3 (4)	C21—C20—C19	120.2 (4)
C10—C9—C8	120.2 (4)	C22—C21—C20	120.2 (4)
C10—C9—H9	119.9	C22—C21—H21	119.9
C8—C9—H9	119.9	C20—C21—H21	119.9
C9—C10—C11	119.7 (4)	C21—C22—C23	119.8 (4)
C9—C10—H10	120.1	C21—C22—H22	120.1
C11—C10—H10	120.1	C23—C22—H22	120.1
C12—C11—C10	120.7 (4)	C24—C23—C22	120.6 (4)
C12—C11—Br1	119.3 (3)	C24—C23—Br2	120.0 (3)
C10—C11—Br1	120.0 (3)	C22—C23—Br2	119.4 (3)
C11—C12—C7	120.7 (4)	C23—C24—C19	120.9 (4)
C11—C12—H12	119.6	C23—C24—H24	119.6
C7—C12—H12	119.6	C19—C24—H24	119.6
			
C1—N1—N2—C2	−0.8 (5)	C14—N6—N5—C13	−0.2 (5)
C2—N3—N4—C6	−178.9 (4)	C13—N7—N8—C18	6.2 (6)
C1—N3—N4—C6	0.5 (6)	C14—N7—N8—C18	−176.1 (4)
N2—N1—C1—N3	0.2 (5)	N6—N5—C13—N7	0.2 (5)
N2—N1—C1—S1	−179.6 (3)	N6—N5—C13—S2	−178.1 (3)
C2—N3—C1—N1	0.5 (4)	N8—N7—C13—N5	177.7 (4)
N4—N3—C1—N1	−178.9 (4)	C14—N7—C13—N5	−0.1 (4)
C2—N3—C1—S1	−179.8 (3)	N8—N7—C13—S2	−4.1 (7)
N4—N3—C1—S1	0.8 (7)	C14—N7—C13—S2	178.1 (3)
N1—N2—C2—N3	1.1 (5)	N5—N6—C14—N7	0.0 (5)
N1—N2—C2—C3	−177.7 (4)	N5—N6—C14—C15	179.8 (4)
C1—N3—C2—N2	−1.0 (5)	C13—N7—C14—N6	0.1 (5)
N4—N3—C2—N2	178.5 (3)	N8—N7—C14—N6	−178.2 (3)
C1—N3—C2—C3	177.8 (4)	C13—N7—C14—C15	−179.7 (4)
N4—N3—C2—C3	−2.7 (6)	N8—N7—C14—C15	2.1 (6)
N2—C2—C3—C4	−5.9 (6)	N6—C14—C15—C16	2.6 (6)
N3—C2—C3—C4	175.5 (4)	N7—C14—C15—C16	−177.8 (4)
C2—C3—C4—C5	179.0 (4)	C14—C15—C16—C17	−178.8 (4)
N3—N4—C6—C7	179.1 (4)	N7—N8—C18—C19	−178.2 (3)
N4—C6—C7—C12	177.3 (4)	N8—C18—C19—C20	1.4 (6)
N4—C6—C7—C8	−3.0 (7)	N8—C18—C19—C24	−179.0 (4)
C12—C7—C8—O1	179.8 (4)	C24—C19—C20—O2	−179.4 (4)
C6—C7—C8—O1	0.0 (7)	C18—C19—C20—O2	0.3 (6)
C12—C7—C8—C9	0.3 (6)	C24—C19—C20—C21	0.7 (6)
C6—C7—C8—C9	−179.5 (4)	C18—C19—C20—C21	−179.7 (4)
O1—C8—C9—C10	179.6 (4)	O2—C20—C21—C22	179.6 (4)
C7—C8—C9—C10	−0.9 (7)	C19—C20—C21—C22	−0.4 (7)
C8—C9—C10—C11	0.3 (7)	C20—C21—C22—C23	0.2 (7)
C9—C10—C11—C12	1.0 (7)	C21—C22—C23—C24	−0.3 (7)
C9—C10—C11—Br1	−177.8 (4)	C21—C22—C23—Br2	−179.6 (3)
C10—C11—C12—C7	−1.6 (7)	C22—C23—C24—C19	0.6 (7)
Br1—C11—C12—C7	177.1 (3)	Br2—C23—C24—C19	179.9 (3)
C8—C7—C12—C11	1.0 (6)	C20—C19—C24—C23	−0.8 (6)
C6—C7—C12—C11	−179.3 (4)	C18—C19—C24—C23	179.5 (4)

### Hydrogen-bond geometry (Å, º)

**Table:**

*D*—H···*A*	*D*—H	H···*A*	*D*···*A*	*D*—H···*A*
O1—H1···N4	0.82	1.93	2.644 (4)	145
O2—H2···N8	0.82	1.90	2.612 (4)	144
N1—H1*A*···S1^i^	0.86	2.46	3.300 (4)	164
N5—H5···S2^ii^	0.86	2.41	3.252 (4)	165

Symmetry codes: (i) −*x*+1, −*y*+1, −*z*; (ii) −*x*, −*y*+1, −*z*.
